# Evolutionary history of *Castanea sativa* in the Caucasus driven by Middle and Late Pleistocene paleoenvironmental changes

**DOI:** 10.1093/aobpla/plad059

**Published:** 2023-08-29

**Authors:** Berika Beridze, Katarzyna Sękiewicz, Łukasz Walas, Peter A Thomas, Irina Danelia, Giorgi Kvartskhava, Vahid Farzaliyev, Angela A Bruch, Monika Dering

**Affiliations:** Institute of Dendrology, Polish Academy of Sciences, Parkowa 5, 62-035 Kórnik, Poland; Institute of Dendrology, Polish Academy of Sciences, Parkowa 5, 62-035 Kórnik, Poland; Institute of Dendrology, Polish Academy of Sciences, Parkowa 5, 62-035 Kórnik, Poland; School of Life Sciences, Keele University, Keele, Staffordshire ST5 5BG, UK; National Botanical Garden of Georgia, Botanikuri Street 1, Tbilisi, Georgia; Faculty of Agricultural Science and Bio-System Engineering, Georgian Technical University, Guramishvili Str. 17, Tbilisi, Georgia; Faculty of Agricultural Science and Bio-System Engineering, Georgian Technical University, Guramishvili Str. 17, Tbilisi, Georgia; Forest Development Service, Ministry of Ecology and Natural Resources, B. Agayev Str, 100 A, Baku, AZ1000, Azerbaijan; The Role of Culture in Early Expansions of Humans (ROCEEH) Research Centre, Heidelberg Academy of Sciences, Senckenberg Research Institute, Senckenberganlage 2560325 Frankfurt/M, Germany; Institute of Dendrology, Polish Academy of Sciences, Parkowa 5, 62-035 Kórnik, Poland; Department of Silviculture, Poznań University of Life Sciences, Wojska Polskiego 71c, 61-625, Poznań, Poland

**Keywords:** Divergence time, genetic diversity, niche modelling, population structure, Early–Middle Pleistocene Transition, refugia, sweet chestnut

## Abstract

Due to global climate cooling and aridification since the Paleogene, members of the Neogene flora were extirpated from the Northern Hemisphere or were confined to a few refugial areas. For some species, the final reduction/extinction came in the Pleistocene, but some others have survived climatic transformations up to the present. This has occurred in *Castanea sativa*, a species of high commercial value in Europe and a significant component of the Caucasian forests’ biodiversity. In contrast to the European range, neither the historical biogeography nor the population genetic structure of the species in its isolated Caucasian range has been clarified. Here, based on a survey of 21 natural populations from the Caucasus and a single one from Europe, we provide a likely biogeographic reconstruction and genetic diversity details. By applying Bayesian inference, species distribution modelling and fossil pollen data, we estimated (i) the time of the Caucasian—European divergence during the Middle Pleistocene, (ii) the time of divergence among Caucasian lineages and (iii) outlined the glacial refugia for species. The climate changes related to the Early–Middle Pleistocene Transition are proposed as the major drivers of the intraspecific divergence and European–Caucasian disjunction for the species, while the impact of the last glacial cycle was of marginal importance.

## Teaser

Sweet chestnut is a remnant of once widely spread ancient Neogene flora, which makes it a relic species. It is a highly economically and ecologically important tree distributed and cultivated mostly in the Mediterranean Basin. The species is also present in the Caucasus region, where it forms natural forests of high conservation value. In this work, we reconstructed the evolutionary history of this species in the Caucasus. We demonstrated that populations retained high genetic diversity and moderate differentiation, partly due to high gene flow and less human impact in the past, in comparison to the European populations. The split between European and Caucasian ranges occurred during the Middle Pleistocene. Based on genetic information, the Caucasian populations of the species were grouped into four genetic clusters which were formed long before the last glacial period.

## Introduction

The early Cenozoic vegetation of the Northern Hemisphere mid-latitudes consisted mainly of mixed mesophytic forests with tropical to warm–temperate taxa, which were widespread during the Paleogene and much of the Early Neogene. However, during the Neogene (the Miocene—23.3–5.33 Ma, and the Pliocene—5.33–2.58 Ma) cooling and aridification occurred and forced pronounced floristic and structural changes in the vegetation in the Northern Hemisphere. Many species of this ancient flora became extinct, and a few survived to become relics that are currently found in several refugia for Neogene species including the Colchic and Hyrcanian forests of the Caucasus (Milne and Abbott 2002).

**Figure 1.**
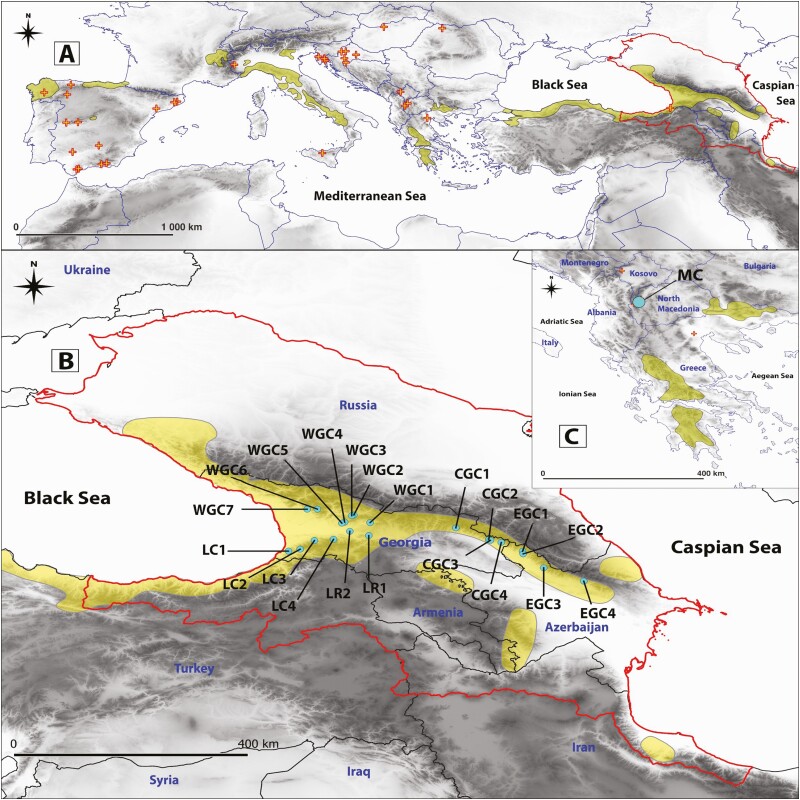
Natural range of *Castanea sativa* (A) according to Caudullo *et al.* (2017), Poljak *et al.* (2017), Martín *et al.* (2012) and Castellana *et al,* (2021); *crosses* denote small and isolated stands of the species; (B) species distribution in the Caucasus with locations of the examined populations on (B) and (C); population acronyms as in Table 1. Eco-regional boundary of the Caucasus is delineated with a solid line.

The Caucasus ecoregion stretches between the Black and the Caspian seas and includes the North Caucasus (Russia), the South Caucasus (Georgia, Azerbaijan and Armenia), north-eastern Turkey and north-west Iran (Fig. 1). The complex geology of the region, resulting from the continuous convergence of the Eurasian and African-Arabian plates has led to high heterogeneity of the landscape and climate, further encouraging the development, accumulation and long-term retention of biodiversity (Tarkhnishvili, 2014). Caucasian forests contribute to the high species diversity and endemism of the region (Tarkhnishvili 2014; Nakhutsrishvili and Abdaladze 2017). As a result, the Caucasus is one of the global hotspots of biodiversity (Mittermeier *et al.* 2011), and yet is endangered by significant habitat loss (Zazanashvili *et al.* 2004), recently further enhanced by anthropogenic climate change (Dering *et al.* 2021; Sękiewicz *et al.* 2022).

The impact of geological and climatic changes on the genetic structure and divergence patterns remains largely unexplored for tree species growing in the Caucasus. The recurring genetic pattern described from the limited studies published to date is the west–east divergence between the Colchic and Hyrcanian populations (Christie *et al.* 2014; Maharramova *et al.* 2015). This genetic differentiation reflects the impact of the two major refugia during the last glaciation located in the Colchic lowland to the west, and Hyrcanian forests to the east (Tarkhnishvili 2014). These refugial areas were the major sources of Holocene re-colonization for plants in the region (Connor and Kvavadze 2009). For some species, the west–east genetic split is also attributed to the influence of the Likhi Range (LR) which longitudinally connects the LC and Greater Caucasus (GC). This mountain range drives the patterns of genetic differentiation and diversity in the region by forming a geographical barrier and influencing the rainfall gradients in the Caucasus (Ekhvaia *et al.* 2018; Sękiewicz *et al.* 2022). However, the derived phylogeographic structures for tree species and level of differentiation are generally weak due to pervasive gene flow (Dering *et al.* 2021; Sękiewicz *et al.* 2022). Comprehensive research in the region is, therefore, needed to fully understand the extent and causes of plant genetic diversity patterns and evolutionary history in the Caucasus, which would help reveal reasons for its extreme biodiversity richness. Additionally, conservation planning and management will strongly benefit from the results of such studies (Beridze *et al.* 2023). Sweet chestnut (*Castanea sativa*) is a thermophilous tree species of high ecological, economic and cultural importance with a complex natural history resulting from domestication (Pollegioni *et al.* 2020; Fernández-López *et al.* 2021). Importantly, it is one of a small number of Neogene relict tree species that have survived in Europe (Dane *et al.* 2003). The species’ natural range extends from the Iberian Peninsula towards the Caspian coast in Iran (Caudullo *et al.* 2017). Sweet chestnut has been extensively studied within its European range (e.g. Krebs *et al.* 2004, 2019; Mattioni *et al.* 2013, 2017; Alcaide *et al.* 2019; Pollegioni *et al.* 2020; Castellana *et al.* 2021; Fernández-López *et al.* 2021), but less so in the Caucasus region (Mattioni *et al.* 2013) despite the Caucasus, along with the neighbouring Asia Minor Peninsula, being the probable initial centre of sweet chestnut domestication and hypothetical source for further dispersal to Europe with cultural and economic exchange between the Middle East and Europe (Pollegioni *et al.* 2020).

Here we investigate the evolutionary history of *C. sativa* in the most eastern domain of its natural distribution—the South Caucasus. Due to the marked geographic isolation and genetic discontinuity between the Caucasian and European populations, the South Caucasus probably host natural populations of *C. sativa* with a unique evolutionary past (Mattioni *et al.* 2017). Moreover, this distinct yet poorly explored gene pool is threatened by the fungal parasite *Cryphonectria parasitica* causing chestnut blight (Tavadze *et al.* 2013), the newly introduced Asian chestnut gall wasp (*Dryocosmus kuriphilus*; Conedera *et al.* 2021), climate change (Conedera *et al.* 2021; Freitas *et al.* 2021), as well as past and ongoing human pressure due to a lack of active forest management (Zazanashvili *et al.* 2004). Thus, our work is an essential step to establish a more complete picture of the natural history of this valuable relic tree species. With the aim of understanding the evolutionary history *C. sativa* in the South Caucasus, we (i) explored the species’ geographic pattern of genetic differentiation, (ii) reconstructed the divergence history of the species, (iii) delineated the location of the probable glacial refugia and (iv) reconstructed the species dynamics of the Holocene expansion in the region.

## Materials and Methods

### Sampling, DNA extraction and genotyping

A total of 21 natural populations of *C. sativa* from the South Caucasus (17 in Georgia and 4 in Azerbaijan) were analysed (Fig. 1, **Supporting Information—Table S1**). In addition, a single population from North Macedonia was included to understand the genetic distinctiveness between European and Caucasian gene pools. A total of 653 mature trees were analysed. DNA was extracted from dry leaves using the CTAB protocol. Nine nuclear microsatellite markers (nSSR)—CsCAT1, CsCAT6, CsCAT14, CsCAT15, CsCAT41 (Marinoni *et al.* 2003) and EMCs2, EMCs13, EMCs15, EMCs22 (Buck *et al.* 2003)—were employed in genetic analyses. PCR conditions were according to Marinoni *et al.* (2003) and Buck *et al.* (2003). PCR (Polymerase Chain Reaction) products were run on Applied Biosystems ABI PRISM 3130 XL genetic analyser (Thermo Fisher Scientific, USA) using an internal standard GeneScan 500LIZ® (Thermo Fisher Scientific, USA). Then, fragment sizes were scored using GeneMapper 4.0 software (Thermo Fisher Scientific, USA).

### Genetic diversity and differentiation

We calculated linkage disequilibrium (LD) in Genepop v 4.7.5 (Rousset 2008) using the log-likelihood ratio statistic test for each pair of loci with Bonferroni correction. Next, the same software was used to calculate deviations from the Hardy–Weinberg equilibrium for each population and each locus. Null allele frequency was estimated using the Maximum-Likelihood method implemented in ML—Null Freq (Kalinowski and Taper 2006). Finally, GenAlEx 6.51b2 (Peakall and Smouse 2012) was used to calculate genetic diversity parameters, including the mean number of alleles (*A*), the number of private alleles (*Pa*), observed heterozygosity (*Ho*) and unbiased estimation of expected heterozygosity (*uHe*). Allelic richness (*Ar*) was estimated in FSTAT 2.9.4 (Goudet 1995).

We tested the statistical difference in parameters of genetic structure (*He*, *Ar*, *Fis* and *Fst* ) between different sets of populations: (i) among populations from the Lesser Caucasus (LC), LR, West Greater Caucasus (WGC), Central Greater Caucasus (CGC) and East Greater Caucasus (EGC) **[see****Supporting Information—Table S1****]** to search for sub-regional patterns; (ii) between Western Georgian populations located in the GC and LC (WGC/LC) excluding populations from the LR (LR1, LR2) to search for diversity pattern specific to different mountain ranges, and (iii) between populations on the west (LC, LR and WGC) and on the east (CGC and EGC) of the region to search for differences in diversity patterns among populations that likely derive from Colchis and Hyrcanian refugial areas, respectively. Statistical significance was calculated in FSTAT 2.9.4 based on 9999 permutations.

Finally, we estimated the inbreeding coefficient (*Fis*) including ‘null alleles’ correction based on the individual inbreeding model using the Bayesian approach implemented in INEst 2.2 (Chybicki and Burczyk 2009). Parameters were specified as 5 × 10^5^ MCMC (Markov Chain Monte Carlo) iterations, storing every 200th value with burn-in steps of 5 × 10^4^. Two independent runs were done for each population, selecting different models according to the Deviance Information Criterion (DIC), that is, the inbred population model (*nfb,* full model where *n* null alleles, *f* inbreeding coefficient, *b* genotyping failure) and the random mating model (*nb*). Then, to estimate the extent of divergence among the Caucasian populations and regions, Wright’s fixation index (*F*_ST_) was calculated using FreeNA with ‘Excluding Null Alleles’ (ENA) correction for the presence of null alleles. The significance of differentiation was tested using bootstrapping resampling over loci method with 10^4^ replications.

### Range-wide population structure

The Bayesian clustering approach implemented in STRUCTURE 2.3.4 (Pritchard *et al*. 2000) was used to infer the genetic structure of the populations in the South Caucasus, and for the whole dataset including the Macedonian stand. The conditions of analysis were as follows: (i) admixture and correlated allele frequencies models, (ii) 10 independent runs for each *K* ranging from 1 to 23 (the whole dataset) or 1 to 22 (the Caucasian populations), and (iii) 10^5^ burn-in periods followed by 1 × 10^6^ MCMC iterations. To infer the optimal number of clusters that best tune with our dataset, the Best *K*, we employed different common approaches (Meirmans 2015). We used Evanno’s Δ*K* approach (Evanno *et al*. 2005), log probability of the data (Ln Pr (*X*|*K*) Pritchard *et al*. 2000), and the algorithm based on the median membership coefficient (*Q*) which appears to be more accurate and reliable than commonly applied methods (Puechmaille 2016). To obtain the *K*-selection plots, we used StructureSelector (Li and Liu 2018), while CLUMPAK (Kopelman *et al*. 2015) was used to summarize and visualize the replicate runs. Being aware of the complexity of factors responsible for structuring diversity, we interpreted the number of genetic clusters in terms of the delivered biogeographic information, as advised (e.g. Meirmans 2015; Cullingham *et al*. 2020). To visualize the spatial genetic structure, the mean membership coefficient (*Q*) values were interpolated across the landscape using QGIS 3.16.3 ‘Hannover’ (QGIS Development Team 2021) using the Inverse Distance Weighting interpolation.

To more deeply explore the spatial pattern of the population differentiation, we used the geogenetic approach implemented in SpaceMix, which allows for the visualization of complex histories of gene flow among populations, including inferring the long-distance dispersal (LDD) of genes and its effect on the genetic structure (Bradburd *et al*. 2016). This approach uses a Bayesian framework to reconstruct the genetic relatedness among populations, shown on a geogenetic map. The geographic location of the populations of such maps is corrected by the impact of the gene flow that increases pairwise genetic similarity (or relatedness). Therefore, population locations on the *geogenetic map* reflect more genetic relatedness rather than only geographic distance (Bradburd *et al*. 2016). Additionally, since the allele frequency covariance is a decreasing function of geogenetic distance, the LDD is manifested as an abnormally strong covariance over large geographic distances (i.e. high genetic similarity between populations despite large distance separating them; Bradburd *et al*. 2016). This method shares the advantages of the non-model approach such as PCA but deals much better with genetic admixture (gene flow) frequently occurring in natural populations. In SpaceMix, we used frequency allele data and tested a model that estimates the population locations and admixture (*source_and_target*). This model locates the studied populations in the geogenetic space independently of their true geographic locations and, at the same time, estimates the gene flow. Simulations were run using 10 initial fast runs, each for 10^5^ MCMC iterations and one long run with 10^6^ iterations with a sampling of every 10^3^. The inferred geogenetic location of the populations and their admixture sources were superimposed on the observed population sampling locations. The uncertainty in the locations of the populations was visualized with 50 % of credible ellipses and inferences about the source of admixture were made for each population to detect the pattern of gene flow and the possibility of LDD. The overall performance of MCMC was validated by exploring the posterior probability trace, while the ability of the model to describe our data was evaluated by comparison of parametric versus observed covariance matrix.

### Demographic history

For a probabilistic analysis of alternative hypotheses for the history of divergence of sweet chestnut lineages from the South Caucasus, we used the approximate Bayesian computation (ABC) procedure combined with supervised machine learning—Random Forest algorithm implemented in R application DIYABC-Random Forest v1.2.1, hereafter ABC-RF (Collin *et al.* 2021). The STRUCTURE’s results **[see****Supporting Information—Figs. S1–S5****]** for the Caucasian stands and the single European one served as a justification for assumptions about the genetic distinctiveness of the North Macedonian site, which was always assumed to be the earliest point of divergence also in tested demographic scenarios. The demographic history of the Caucasian populations was based on STRUCTURE’s *K* = 4 performed for this set of populations (see Results). For dating the divergence points among genetic lineages, we selected individuals that showed at least 80% of ancestry to the respective cluster, hereafter called a lineage. As a result, the gene pool representing the LC (Lineage I) consisted of 62 individuals, the gene pool of the WGC (Lineage II) consisted of 72 individuals, the CGC (Lineage III) consisted of 65 individuals, and the population EGC4 that represents the most easterly located and most genetically distant Caucasian gene pool consisted of 40 individuals (Lineage IV). The European gene pool consisted of 27 individuals (Lineage V).

Eight scenarios regarding the topology of population ancestry were constructed based on the patterns of genetic differentiation revealed by STRUCTURE (Fig. 4). Based on the results of the phylogenetic studies (Janfaza *et al.* 2015), we made an assumption about the common ancestor for all genetic lineages investigated here. The two first scenarios included an admixture event, while the remaining six scenarios presented divergence events of different hierarchies. Scenario 1 assumed the first split at *t2* generations ago among Lineage I (LC), Lineage IV (EGC—population ECG4) and Lineage V (Europe). Further, at *t1* generations ago, Lineage II (GC) diverged from Lineage I. The last event included the admixture *ta* generations ago between Lineages II and IV that resulted in Lineage III. Scenario 2 was a modification of Scenario 1 in the way that it assumes admixture between the same lineages but without any previous divergence of Linage II from Linage I. Scenario 3 presented the differentiation of all genetic lineages throughout a single divergence event *t1* generations ago. Scenario 4 refers directly to the *K* = 4 structure and west–east differentiation and shows that Lineages 2 and 4 diverged from Lineages 1 and 3, respectively. Scenarios 5–8 assumed several topologies that describe different hierarchies of divergence and ancestry for the current lineages.

We used a 2 × 10^4^ simulated training data set per scenario for model selection at the first step of ABC-RF analysis. After testing, the number of trees in the constructed random forests was set to 2000 as this was large enough to ensure a stable estimation **[see****Supporting Information—Fig. S9****]**. Next, the most likely scenario was evaluated based on a classification vote for each scenario, representing the number of times a scenario is selected in a forest of *n* trees and the posterior probability estimator. The compatibility between scenarios and/or associated priors and the observed data was assessed using the linear discriminant analysis (LDA). After processing the scenario of choice, the second step of ABC-RF analysis included parameter estimation for the most likely scenario. To do this, we performed independent ABC-RF treatments for each parameter of interest based on an extended training data set as 10^5^ simulated datasets, 2000 trees with 1000 out-of-bag samples used as a test, five axes of PLS (partial least squares regression analysis) derived from the PLS processed on the summary statistics, and summarized using the 130 statistics that described the genetic diversity within the populations, between pairs or per triples of populations, averaged over all loci used. We inferred the mean and the median as point estimates for each parameter and computed global and local accuracy metrics corresponding to global and local Normalized Mean Absolute Error and the 90% coverage.

To evaluate whether our training set is sufficient for ABC-RF analysis, we applied procedures recommended by Collin *et al.* (2021) to compare the accuracy metrics’ stability under different subsets of the training dataset. The out-of-bag prediction method (Pudlo *et al.* 2016) based on a 1000 out-of-bag dataset was used to evaluate the prediction accuracy of the scenario choice and parameter estimations based on global and local errors. The global (i.e. prior) error indices correspond to prediction quality measures computed over the entire dataset. In contrast, the local (i.e. posterior) error indices that are conditionally computed on the observed dataset refer to the prediction quality precisely at the position of the observed dataset. For the scenario choice, we conducted ten replicate ABC-RF analyses based on the same training dataset (Collin *et al.* 2021). Analysis was conducted with the Stepwise Mutation Model with two parameters: the mean mutation rate (*µ*) and the mean parameter of the geometric distribution (*P*) used to model the length of mutation events. We assumed uniform distribution for the mean mutation rate and *γ* for the remaining genetic parameters; their ranges are shown in **Supporting Information—Table S2**.

ABC-RF analysis provides the time from the demographic event (split or merging) in the number of generations that needs to be converted into absolute time to make a correlation with possible causal factors (paleoclimate evolution, geological events, etc.). This stage of the analysis is prone to bias due to the unknown true generation time for most species and therefore requires careful consideration. The generation time used in this study is a proxy for the true generation time, which has not yet been established for sweet chestnut. However, we prefer not to use the age at first reproduction applied by some investigators as a surrogate for generation time in trees, which are long-lived species. Age at first reproduction would significantly bias the results by underestimating the true generation time and consequently underestimating the time since common ancestry and other demographic events. Hence, a generation time for *C. sativa* of 100 years was used, similar to Fernández-López *et al.* (2021) in reconstructing the demographic history of the species in the Iberian Peninsula in order to maintain comparability between the results of that study and our investigations. A similar generation time has been assumed for other long-lived tree species in the studies of their demographic history using the ABC approach (Mayol *et al.* 2015; Pollegioni *et al.* 2017).

### Niche modelling and paleobotanical records

The theoretical range of *C. sativa* in the past was estimated to support our demographic estimations and track the location of potential glacial refugia for the species in the Caucasus. For this purpose, we employed the Species Distribution Modelling (SDM) approach based on the maximum entropy algorithm implemented in MAXENT 3.4.3 (Phillips *et al.* 2004). We determined the species distribution in the South Caucasus for the following periods: the Last Interglacial (LIG, ca. 140–120 ka), the Last Glacial Maximum (LGM, ca. 21–17 ka), the Early Holocene (EH, 11.7–8.326 ka), the Middle Holocene (MH, 8.326–4.2 ka) and the Late Holocene (LH, 4.2–0.3 ka).

First, our study area was defined within latitudes from 35.77 to 45.16 and longitudes from 38.35 to 56.00. Initially, 214 georeferenced occurrence spots were collected but after data treatment in QGIS to meet the uniform coverage to the study area, in total, 92 unique points were delivered for further SDM analysis. Next, a set of bioclimatic variables (Bio1–Bio19) at 30 arc-sec resolution for the current period (1979–2013) were downloaded from CHELSA 1.2 (Karger *et al.* 2017). The same set of climatic variables was obtained from the PaleoClim for the past periods, that is, LIG, LGM, EH, MH and LH (Otto-Bliesner *et al*. 2006; Fordham *et al.* 2017; Brown *et al.* 2018) with a 2 arc-min resolution. To avoid the multi-collinearity among variables in the studied landscape, the *vif* function implemented in the *usdm* R package was used (Naimi *et al.* 2014). Finally, seven variables (Bio1—Annual Mean Temperature, Bio3—Isothermality, Bio8—Mean Temperature of Wettest Quarter, Bio9—Mean Temperature of Driest Quarter, Bio15—Precipitation Seasonality, Bio18—Precipitation of Warmest Quarter, Bio19—Precipitation of Coldest Quarter) remained for further analyses. MAXENT was run with a “random seed” option, in which 20 % of the occurrence was set as test data for model evaluation. Replicates were specified as 100 for bootstrap procedures with maximum iterations of v10^4^ and output was set as logistic. The area under the curve (AUC) values were used to estimate the performance of each model (Phillips *et al.* 2004; Wang *et al.* 2007). The high values of AUC (>0.9) indicate a significant prediction power; SDM results were visualized in QGIS.

To support our SDM findings, we used data on pollen and macroremains records from Krebs *et al.* (2019). We collected and filtered records to match the Caucasian ecoregion. Then, we overlaid the occurrence of pollen and macroremains on the maps delivered from MAXENT analysis showing the suitability areas in LGM, EH, MH and LH using QGIS. Due to the history of cultivation and human influence on the distribution of *C. sativa*, it was crucial to divide pollen records into groups that accounted for the probability of human-induced dispersion of the species in the South Caucasus. Therefore, based on Krebs *et al.* (2019), we divided the collected data for the LH period into pre-cultivation records (up to 2500 BP) and cultivation records (after 2500 BP). In total, 89 records were retrieved from Krebs *et al.* (2019), of which 9 were solely macroremains. The pre-cultivation period consisted of 58 records, while 31 were ascribed to the cultivation period.

## Results

### Genetic diversity

All analysed nSSR loci were neutral, and no LD between each pair of loci was detected. Null alleles were presented in all loci with a frequency ranging from 0.009 (EMCs13) to 0.120 (CsCAT41) and an average value of 0.004 **[see****Supporting Information—Table S3****]**. A total of 113 different alleles were found in studied populations ranging from 5 (EMCs2) to 25 (CsCAT41), with a mean value of 12.55 per population.

The highest average number of alleles per locus (*A*) was noted in the two LC populations (6.67, LC1 and LC4) and the lowest in the CGC and EGC (3.56, CGC4 and EGC3). This pattern was also found in the private alleles frequency, where the highest Pa (1.75) was noted in LC populations and the lowest (0.25) in EGC. Observed heterozygosity (*Ho*) ranged from 0.583 (WGC5) to 0.413 (CGC3), while populations from the LC presented the highest level of *uHe*, reaching 0.594 in LC2, and the lowest was observed for the CGC stand (0.415 in CGC4). The highest *Fis* was observed in LC4, WCG6 and CGC3, reaching 0.111, 0.131 and 0.136, respectively. The remaining populations had relatively low values and remained below 0.1. According to DIC, the homozygosity excess in the populations with the highest *Fis* was likely due to inbreeding. See **Supporting Information—Table S1** for details on the genetic structure of the population.

The global genetic differentiation among populations was moderate, reaching 0.076 (95 % CI: 0.055–0.094), while the *Fst* estimation with the ENA correction was slightly lower *Fst = *0.073 (95% CI 0.052–0.092). Pairwise *Fst* ranged from 0.004 (WGC4/WGC3) to 0.266 (EGC4/CGC4). Generally, the most divergent was the most easterly located population, EGC4, which attained the highest values in pairwise comparisons and likely, the distinctiveness of this population affected the overall level of differentiation **[see****Supporting Information—Table S4****]**.

The regional-level analysis showed statistically significant differences in *Ar* and *He* among the groups of populations in three scenarios out of four tested **[see****Supporting Information—Table S5****]**. No differences were found in any parameters of the genetic structure between populations from the LC and WGC. The global differentiation (*Fst*) among the groups of populations was significant only when comparing West Caucasian populations (LC, LR and WGC) to Central and East Greater Caucasian populations (CGC, EGC), reaching mean values of 0.046/0.095, respectively (*P* < 0.0369); no statistical difference was observed in *Fis* values **[see****Supporting Information—Table S5****]**.

### Range-wide population structure

An optimal number of clusters for the South Caucasus populations and the North Macedonian site was *K* = 3 according to the Δ*K* method, and *K* = 10 in the case of LnP(*K*) (Mean LnP(*K*) = −13 694.3; SD = 12.63179), while the approach of Puechmaille (2016) retrieved five clusters from the data **[see****Supporting Information—Figs. S1–S5****]**. Both clustering approaches underpinned the unique genetic composition of the Macedonian population. Furthermore, the clustering at *K* = 5 delivered the same differentiation pattern as an analysis conducted for the South Caucasus subset of populations described below (Figs. 1 and 3).

**Figure 2.**
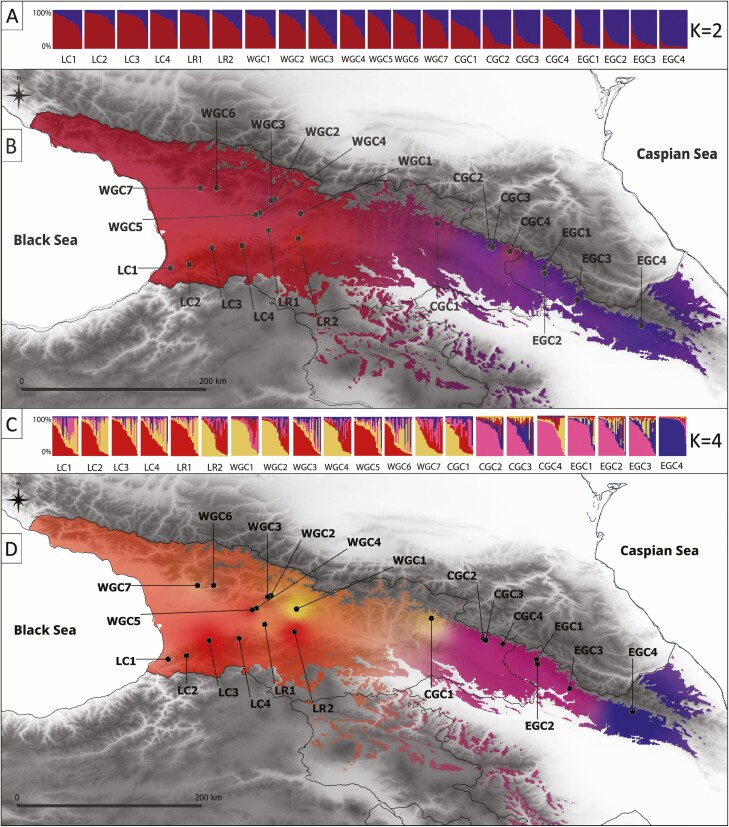
Spatial genetic structure of populations of *Castanea sativa* in the South Caucasus inferred by STRUCTURE: (A) barplot of individual estimated membership using *K* = 2; (B) interpolation results throughout the landscape at *K* = 2 based on *Q*-membership values; (C) barplot of individual estimated membership using *K* = 4, (D) interpolation results throughout the landscape at *K* = 4 based on *Q*-membership values; interpolation results were visualized using QGIS; population acronym as in Table 1.

Based on LnP(*K*), the optimal number of homogenous groups in the Caucasian dataset (21 populations) was *K* = 10 (Mean LnP(*K*) = −13 185.36; SD = 140.42), which was too high to provide any other explanation but the idiosyncratic history of each population **[see****Supporting Information—Fig. S6****]**. In contrast, Evanno’s Δ*K* method applied to the Caucasian populations (Georgia and Azerbaijan) produced an optimum *K* = 2 **[see****Supporting Information—Fig. S7****]**. This grouping pattern assumed Western (Cluster I: WGC and LC) and Eastern (Cluster II: CGC and EGC) gene pools and clearly depicted admixture between them (Fig. 2A and B). The population CGC1 located between those two gene pools exemplified this genetic admixture as it displayed almost equal average membership (*Q-*value) to both clusters (Cluster I: 52% and Cluster II: 48%). However, an outlier from the general pattern was the CGC4 population, with a high proportion of Cluster I (56%) but located in the region of Cluster II.

Further details in the genetic structure were delivered by using *K* = 4, based on the method of Puechmaille (2016) (Fig. 2C and D, **Supporting Information—**Fig. S8). Generally, *K* = 4 indicates sub-structuring in the western and eastern parts of the species range, also revealing a more complex pattern of genetic structure. Specifically, the populations from the LC (LC1–LC4) were grouped in Cluster I together with two from the WGC (WGC3 and WGC5) and one from the eastern edge of the LR (LR1), while Cluster II had wider spatial distribution and consisted of three populations from the WGC (WGC1, WGC2, WGC4), LH2 from the western edge of the LR and single population from the CGC (CGC1). However, the majority of populations in both mountain ranges display a relatively high admixture with no predominance of any of the two clusters. This is well exemplified in populations WGC6, WGC7 and CGC1, which possessed almost equal *Q*-values to Clusters I and II. The highest *Q*-value of Cluster I was in population LC3 (61.8%), while Cluster II—WGC1 (61.4%). Cluster III was defined for populations distributed in the CGC (CGC2–CGC4) and the EGC (EGC1–EGC3). Here, we also noted ca. 20% of the genomes were attributed to Clusters I and II. At the easternmost part of the studied area, the population EGC4 of Cluster IV shows a high divergence from the other populations studied, confirmed by the highest values of pairwise *Fst* reaching >10%. The admixture of this distant genetic pool at 24.5 % and 22.4 % were detected in EGC3 and EGC2, respectively.

**Figure 3.**
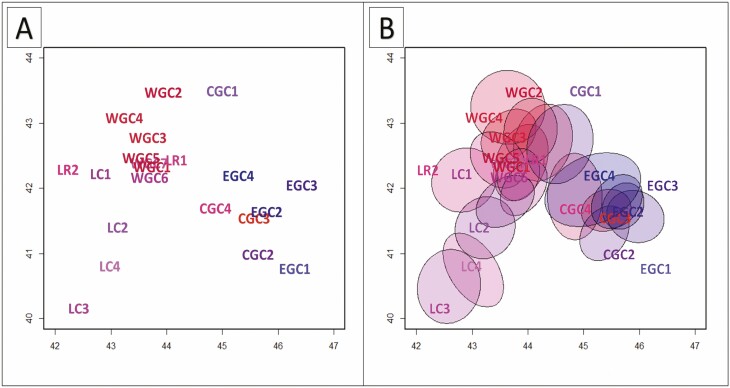
The location of studied populations of *Castanea sativa* in geogenetic space without (A) and with (B) the 50% uncertainty (ellipses) in current geographic locations due to gene flow (SpaceMix analysis). The *X* and *Y* axes present longitude and latitude, respectively; populations acronym as in Table 1.

The constructed geogenetic map of population relationships made by SpaceMix is given in Fig. 3. Plots of the posterior probability trace and model adequacy indicated that MCMC mixed well and achieved the acceptance rate close to the desired 44 %, and the model adequately described the data. The SpaceMix analysis recovered the spatial pattern of two major groups that have been revealed by STRUCTURE’s *K* = 2 with the intermediate position occupied by CGC1 (Fig. 3A). SpaceMix primarily shows the west–east spatial dimension of the divergence, but it also suggests a north–south pattern that reflects some distinctiveness between the GC and LC. The populations’ uncertainty in location shows that they are generally located in one of the two groups. However, for some populations, their geogenetic locations are significantly pulled away from their current geographic one (e.g. CGC1, CGC2, EGC1 and EGC3), which likely reflects genetic relatedness with other populations and long-distance gene flow (Fig. 3B). On the other hand, their geogenetic locations are closer for some populations despite the considerable geographic distance. The detailed insight into the admixture pattern reveals persuasive gene flow and variability of admixture in terms of direction. For example, population CGC1 reflects substantial admixture from the geogenic space related to the LC, while LR2 to the GC (Fig. 3B).

### Demographic history

The classification votes and posterior probabilities estimated for the observed microsatellite dataset in all 10 replications of ABC-FR analyses were the highest for Scenario 1 having an average of 574 (SD = 31.522) votes out of 2000 RF-trees; the average posterior probability was PP = 0.708 (SD = 0.035). The global probability of incorrectly identified scenarios (prior error rates) in tested datasets was estimated to be 0.23942, and the highest-class error rates were in Scenarios 7, 6 and 2 (0.355, 0.340 and 0.288, respectively). For Scenario 1, the posterior (local) error rate was, on average, 0.238 (SD = 0.0006). The fit of Scenario 1 to the observed dataset is illustrated by the projection of the simulated and observed datasets on the first two LDA axes **[see****Supporting Information—Fig. S9****]**. The second-best scenario obtained 280 (SD = 34.693) votes and it was Scenario 8 in which Linage 4 was ancestral to all Caucasian lineages.

**Figure 4.**
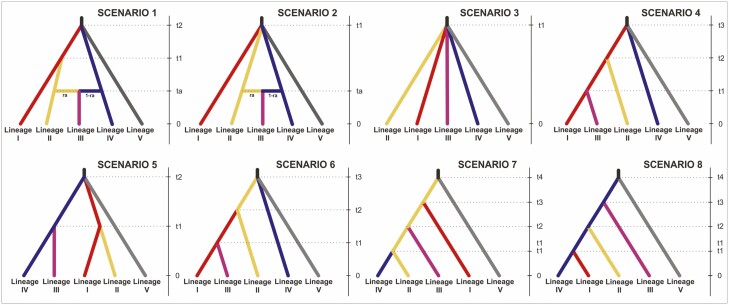
Eight demographic scenarios of the possible evolutionary history of *Castanea sativa* tested in the ABC-RF analysis. The estimated time of demographic and admixture events were scaled by the species generation time (100 years). Genetic lineages representing the main clusters inferred by STRUCTURE, that is, Lesser Caucasus (Lineage I), Greater Caucasus, (Lineage II), West + East Greater Caucasus (Lineage III), East Greater Caucasus (Lineage IV) and Europe (Lineage V).

Under the most likely Scenario 1 (Fig. 4), at *t*2 = 497.3 ka BP (95% CI: 243.1–856.7 ka BP), the ancestor lineage split into Lineages I, IV and V, which is primarily translated into a disjunction between the Caucasian and European gene pool. At *t*1 = 150.3 ka BP (95% CI: 79.2–195.6 ka BP), Lineage II, with the highest frequency in WGC, diverged from Lineage I which predominates in the LC. Finally, the admixture between Lineages II and IV dated at *ta* = 90.8 ka BP (95% CI: 29.4–157.7ka BP) led to Lineage III. The highest effective population size was estimated for Lineage I (Ne = 15 322), and the lowest for Lineage III (Ne = 2698, Table 1).

**Table 1.** Parameter estimates for the best demographic Scenario 1 tested in ABC-RF. Ne, effective ancestral population size; *ta*, time of admixture event; *ra*, level of admixture; *t*1–*t*2, time-of-split events; *µ*mic, mutation rate.

Parameter	Median	95% CI
Ne Lineage I Lesser Caucasus	15 322	9293–19 620
Ne Lineage II Greater Caucasus	4629	2160–8274
Ne Lineage III West + East Greater Caucasus	2968	1222–6457
Ne Lineage IV East Greater Caucasus	4619	2571–6688
Ne Lineage V Europe	7438	3713–13 600
*ta*	90 800	29 400–157 700
*ra*	0.71	0.37–1.14
*t*1	150 300	79 200–195 600
*t*2	497 300	243 100–856 700
*µ*mic	4.07 × 10^−6^	3.85 × 10^−7^–9.29 × 10^−9^

### Niche modelling and paleobotanical records

The MAXENT model accuracy (AUC) for all presented SDM models was greater than 0.93 **[see****Supporting Information—Table S7****]**. Depending on the model, the most important variables in the SDMs were the precipitation of the warmest quarter (Bio18) and the precipitation of the coldest quarter (Bio19), with a relative contribution reaching >54 % and >21 %, respectively. In addition, the annual mean temperature (Bio1), with a relative contribution of >11%, also had a significant impact on the projected species distribution in all models.

**Figure 5.**
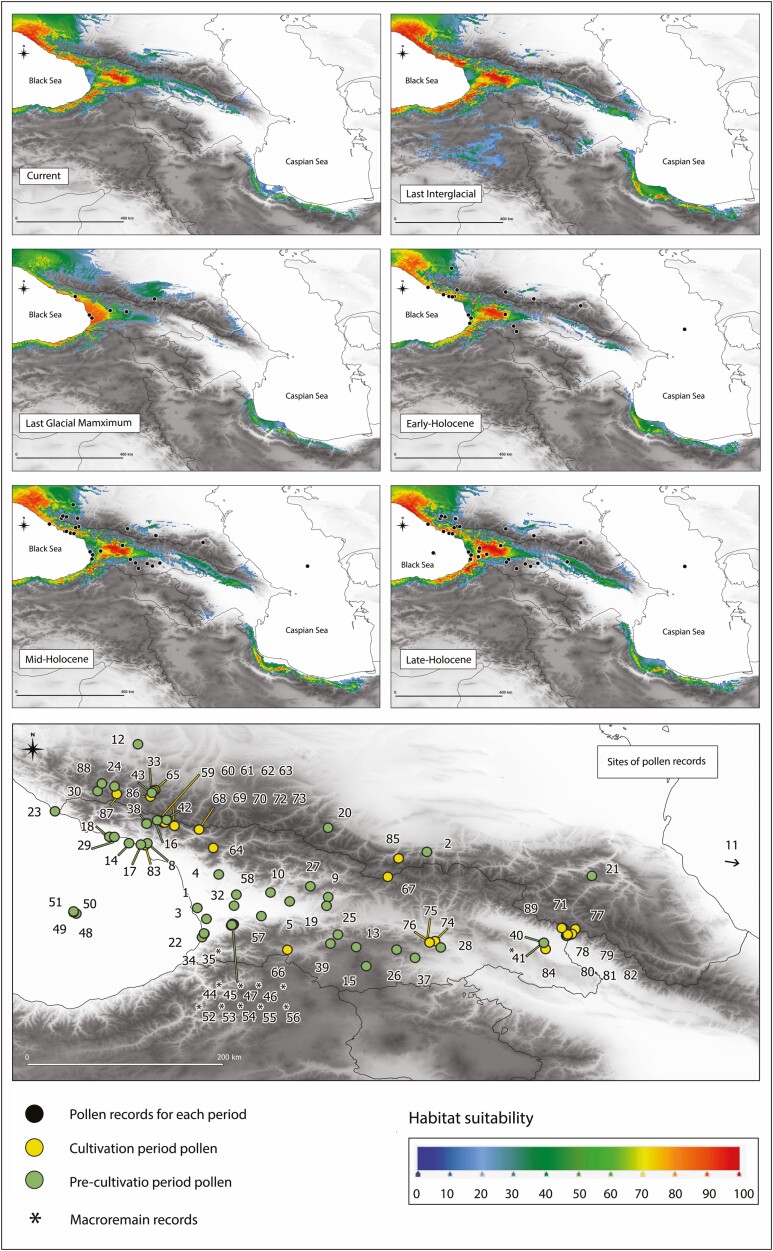
Estimation of the potential range of *Castanea sativa* occurrence in present times (1979–2013), the Last Interglacial (LIG, ca. 130 ka), the Last Glacial Maximum (LGM, ca. 21–17 ka), the Early Holocene (EH, 11.7–8.326 ka), the Middle Holocene (MH, 8.326–4.2 ka) and the Late Holocene (LH, 4.2–0.3 ka), with pollen record sites from the corresponding periods taken from Krebs *et al.* (2019). Sites of pollen record represent the cultivation period (up to 2500 BP) and the pre-cultivation period (after 2500 BP). SDMs ware visualized using QGIS.

The potential distribution of *C. sativa* across the studied landscape in different time slices is given in Fig. 5. The current theoretical range overlaps with the known current species distribution. Regarding niche suitability, the most suitable habitats were in the westernmost part in the foothills of the WGC, Abkhazia and East Pontic Mts. in Turkey. Towards the east, habitat suitability declines to a maximum value of 20–40% in the CGC and EGC (Azerbaijan). The second domain of the potential distribution covers Hyrcanian forests in Iran, where the species is currently limited to a few stands in the west. However, a higher potential suitability of 50–70% compared to the eastern part of the GC was projected for sweet chestnuts in the Hyrcanian forest.

The niche model for LIG indicates that the species covered approximately the same areas as the current range. Additionally, MAXENT modelled small pockets of the species occurrence in the Azerbaijan part of the GC (suitability up to 60 %), the LC (up to 40 %), central Armenia and southern territories of Turkey where the species is not present currently. The projected distribution in Hyrcania in LIG is broader than currently, with much higher suitability. There was a clear distributional gap between Hyrcanian forests and the GC in that period.

The modelled distribution contracted significantly at the LGM. Suitable climatic conditions were projected primarily in Colchis Lowland near the Black Sea and in a narrow strip of coastal areas close to the Pontic Mts. The Hyrcanian forest had relatively high suitability in LGM for *C. sativa* (40–60%), as did the North Caucasus (Kabardino–Balkaria–North Ossetia, suitability ca. 50 %. The model for the EH suggested an expansion of the distribution from the Colchic refugium to the east and possibly an expansion in the Hyrcanian forest. In the MH, the range remained more or less the same as in EH in terms of the area, but habitat suitability in eastern locations increased. The only noticeable difference by the LH was lower suitability values in the Hyrcanian forest compared to MH, but still higher than it is today.

The compiled fossil pollen records retrieved from Krebs *et al.* (2019) are largely in line with the modelled species range from the LGM to the current times in terms of the projected distribution in each analysed period (Fig. 5). The data support the modelled presence of *Castanea* during LGM in areas indicated with moderate (50 %) and high (90–100%) climatic suitability. For EH, the fossil pollen of sweet chestnut was reported in cores taken from sites beyond the projected range, that is, in the Javakheti Plateau and some locations in the Northern Caucasus, undoubtedly a result of long-distance pollen transport. Similarly, the cores from the Caspian Sea containing sweet chestnut pollen refer to wind transportation of pollen from the Hyrcanian forests.

## Discussion

### The European–Caucasian divergence

Our demographic reconstructions suggest that the genetic split between the European and the Caucasian gene pools of *C. sativa* occurred during the Middle Pleistocene (Fig. 4; *t*2* = *497.3 ka, 95% CI: 243.1–856.7 ka). This corresponds to the Early–Middle Pleistocene Transition (EMPT) 1.2–0.6 Ma (Head and Gibbard 2015). The weaker and shorter glacial cycles with a ~40 000 years frequency that predominated in the first ca. 1.5 million years of the Pleistocene gave way to longer and stronger oscillations lasting 100 000 years. Consequently, the glaciations after EMPT were generally longer, colder and drier, leading to significant vegetation turnover (Chalk *et al.* 2017). Paleoecological reconstructions indicate a significant drop in temperatures in the West Caucasus at the beginning of the Middle Pleistocene (Mahler *et al*. 2022). In Europe, the glacial maxima during the Middle Pleistocene (especially MIS 16 and MIS 12) contributed to the final reduction of *Castanea* in forests (Tzedakis *et al.* 2006). The progressive disappearances of several other tree species as a response to EMPT are reported in pollen records (Magri and Palombo 2013). Indeed, the divergence between the Alpine and Iberian lineages of the *Abies alba* was attributed to the impact of EMPT (Scotti‐Saintagne *et al.* 2021). Thus, our results are in line with other studies suggesting a large-scale impact of EMPT on biogeographical patterns not only in Europe but also in West Asia.

In our demographic reconstruction, we used a single population from Macedonia to represent the European genepool of sweet chestnut. Such a sampling scheme is a methodological caveat that has to be addressed due to potential bias. The most recent study (Fernández-López *et al.* 2021) has shown that the Early–Middle Pleistocene was the time of the final fragmentation of the otherwise continuous range of *Castanea* in Europe and the divergence between the West Mediterranean (Iberian) and East Mediterranean (Greek) chestnut genepools. Similarly, our result indicates that the European and Caucasian lineages diverged due to the EMPT. Despite the limited sampling of the European range, our analysis has captured the signal of the serious transformation of the sweet chestnut distribution in Europe and Western Asia as a result of paleoenvironmental change following the EMPT.

### The Middle to Late Pleistocene genetic splits and admixture in the Caucasus

The strong signal of east–west divergence in the sweet chestnut Caucasian gene pool was confirmed by two different analyses of our dataset. First, the STRUCTURE analysis revealed two clusters (*K* = 2) that captured the major structure in the data (Fig. 2A and B). Next, the SpaceMix approach (Fig. 3) repeated this east–west split in genetic structure. Similar genetic patterns, indicating a pervasive West–East divergence, have been found in other Caucasian relic tree species (Christie *et al.* 2014; Maharramova *et al.* 2015).

Clustering at *K* = 4 (Fig. 2C and D) clearly differentiated the marginal population ECG4 in Azerbaijan. This genetic distinctiveness was further confirmed in pairwise comparisons by high values of *Fst* (11.6–26.6%, **Supporting Information—Table S4**). The best demographic Scenario 1 (Fig. 4) suggests that the split between Lineage IV, represented by ECG4, and Lineage I (the LC), was as old as the European–Caucasian divergence and occurred in the Middle Pleistocene (497.3 ka). Accordingly, the east–west genetic split in *C. sativa* in the Caucasus may have been caused by profound changes in the glaciations that occurred in the Northern Hemisphere after the EMPT (Fernández-López, *et al* 2021).

Scenario 1 assumes the split between Lineage I (the LC) and Lineage II (the GC) occurred in the second part of the Middle Pleistocene (Fig. 4; *t*1* = *150.3 ka BP; 95% CI: 51.8–189.9 ka BP), which represents the penultimate glacial of MIS 6 (Krijgsman *et al.* 2019). During this glacial period, *Castanea* pollen is sporadically recorded, suggesting a likely range contraction and fragmentation, both of which may cause genetic divergence. This severe glacial period has been recently shown to play a key role in the evolution and distributional pattern of the herbaceous *Bunias orientalis* in Europe and the Caucasus (Koch *et al.* 2017).

The last demographic event in the evolutionary history of sweet chestnut in the Caucasus identified by Scenario 1 is the merging of eastern Lineage IV and western Lineage II (Fig. 4), which gave rise to Lineage III. The populations belonging to the gene pool of Linage III (CGC2–EGC3) are now distributed in the EGC and were defined by STRUCTURE as a separate cluster (Fig. 2C and D). According to demographic reconstructions, the admixture between both lineages likely happened at *ta = *90.8 ka BP (Fig. 4; 95% CI: 29.4–157.7 BP), which coincides with the beginning of the last glacial period in the region and correlates with MIS 4 (Krijgsman *et al.* 2019). The admixture was asymmetrical, and Lineage II mainly contributed (*ra* = 0.71, Table 1). Typically, in the demographic history of species, the admixture event coincides with the range expansions and overlaps that occur during the re-colonization period (e.g. Havrdová *et al.* 2015). For sweet chestnut in the Caucasus, this could occur as a response to glaciation. This assumes a shift of some eastern range populations (Linage IV) towards a more humid west, and mixing with populations belonging to Lineage II. However, the broad range of this event includes LIG. In this case, the admixture could also be probable due to forest expansion in LIG recorded by pollen data (Mahler *et al*. 2022). Therefore, the timing and drivers of admixture between Lineages II and IV require caution. Further sampling in the eastern part of the region (Azerbaijan), in Hyrcania and also in individual stands in Armenia could help to resolve this uncertainty.

### Refugia during the last glacial cycle and current diversity patterns

Accurately assessing areas of species refugia requires integration of information since approaches based on fossils, genetic data or SDMs are each subject to limitations and biases (Gavin *et al*. 2014). In the Caucasus, pollen data indicated the location of major refugia in Colchis and Hyrcania, which is supported by SDMs and genetic studies (Tarkhnishvili *et al.* 2012; Aradhya *et al.* 2017; Volkova *et al.* 2020; Sękiewicz *et al.* 2022). According to our SDM analysis, *C. sativa* could have survived LGM mostly in the Colchis Lowland and partly in the eastern Pontic Mts. and the Hyrcanian forests. Generally, glacial refugia are expected to harbour higher levels of genetic variability, especially allelic richness and private alleles. In our study, this was found in populations from the western LC (populations LC1–LC4), which display the highest gene diversity and number of private alleles **[see****Supporting Information—Table S1****]**. Additionally, the genetic lineage from this area (Lineage I) had the largest ancestral effective population size as estimated by demographic analysis (*N*1 = 15 322, Table 1). Our niche modelling suggests that sweet chestnut survival during the glacial cycle was also theoretically possible in the North Caucasus (Ingushetia and Chechnya) and in the EGC, Dagestan and Azerbaijan, where the species was likely also present in LIG (Fig. 5). In these refugia, the ancestral populations of current Linage IV likely persisted in the glacial conditions. Paleobotanical records from the Khunzakh Plateau (southern Dagestan, bordering on Azerbaijan to the north) show *C. sativa* from EH deposits (9200–8980 cal BP; Ryabogina *et al.* 2019), suggesting local presence. A better representation of this region with genetic and paleobotanical data would help to prove this hypothesis.

Our results show that the EH range of *C. sativa* projected by MAXENT is comparable in size and suitability to the current theoretical range, except for the eastern parts of the region (Fig. 5). According to paleobotanical evidence, the colonization of the most eastern part of the region by trees was hampered by a relatively dry climate at the beginning of the Holocene, especially during spring months (Messager *et al.* 2021). Currently, stands of sweet chestnut in the eastern areas of the Caucasus (EGC1–EGC4) display the lowest values of gene diversity **[see****Supporting Information—Table S1****]**. The strong bottleneck effect during colonization of these areas and exposure to drift in much harsher habitats may be a reason for observed genetic impoverishment in these populations of sweet chestnut **[see****Supporting Information—Table S1****]**. This explanation has been used for the populations of *Quercus petraea* subsp. *iberica* growing in the eastern part of the Caucasus (Ekhavaia *et al.* 2018).

Despite the rugged landscape of the Caucasus, the STRUCTURE (Fig. 2) and SpaceMix results (Fig. 3) both revealed intensive gene flow among populations of sweet chestnut, similar to other tree species studied in the region (Maharramova *et al.* 2015; Dering *et al.* 2021; Sękiewicz *et al.* 2022). Both methods showed the prevailing gene exchange was between sweet chestnut populations located in western parts of the LC and GC (Fig. 2 and 3); lower gene flow was indicated among the populations from CGC and EGC. This may point to the LR as a significant biogeographic barrier preventing wide genetic connectivity which was previously detected for *Q. petrea* subsp. *iberica* (Ekhvaia *et al.* 2018). Nevertheless, the gene flow in *C. sativa* remains quite intensive, ascribed to the relatively small dimensions of the studied area and the potential of long-distance pollen dispersal in species (Larue *et al.* 2021). Although predominantly insect-pollinated, sweet chestnut may use wind in pollination (Larue *et al.* 2021). The homogenizing effect of gene flow is reflected in a moderate level of differentiation among studied populations, reaching 7 %. The level of admixture in Europe is more limited, resulting in a clear geographic structure, and differentiation reaching 15 % (Mattioni *et al.* 2013; Castellana *et al.* 2021). Additionally, the results of SpaceMix underlined the complexity of the coalescence process involving inherent temporal and spatial aspects (Bradburd and Ralph 2019). Our results indicate the impact of gene flow, especially long distance, on the genetic composition of populations. For that reason, some uncertainty (denoted as ellipses in Fig. 3) was inferred between the current geographic locations of the populations, their genetic composition and source of gene flow.

### Final remarks

In contrast to the European gene pool of sweet chestnut that has been substantially altered by cultivation (Martín *et al.* 2012; Fernández-López *et al* 2021), the use of sweet chestnut in the Caucasus for timber, nuts, fodder and honey production was without large-scale commercial planting and was restricted to small orchards using local seeds (Bobokashvili and Maghradze 2009). During the Soviet period, attempts to cultivate the species were undertaken in the North Caucasus (Abkhazia, Russia) but failed due to fungal and bacterial blight outbreaks (Pridnya *et al*. 1996). However, severe and uncontrolled cutting of the tree occurred in Georgia during the 19th century due to high European demands for sweet chestnut timber, which led to the highly fragmented distribution (Bobokashvili and Maghradze 2009). Consequently, the biogeographic patterns uncovered for sweet chestnut in the Caucasus are likely less disturbed than in Europe and more likely to reflect natural evolutionary changes. Indeed, there is no detectable increase of sites with *Castanea* pollen after 2500 BP (Krebs *et al.* 2019), which would have suggested a spread of the species by humans (Fig. 5).

Most of today’s populations of sweet chestnut are seriously fragmented and a mass die-off of trees is observed (Tavadze *et al.* 2013). Due to sweet chestnut’s longevity, the adverse effects of this are not yet detectable—populations display generally high gene diversity and low inbreeding. Although the regional impact of future climate change in the Caucasus is not yet fully understood, especially regarding precipitation changes (IPCC 2014, Figure SPM.7), it will likely amplify the biotic stress on sweet chestnut.

## Supporting Information

The following additional information is available in the online version of this article –

**Table S1**. Population identity, location, and genetic diversity parameters for studied populations of sweet chestnut from the South Caucasus and North Macedonia

**Table S2.** Priors used for ABC procedure in DIYABC-Random Forest for demographic history inferences for *C. sativa*

**Table S3.** Characterization of nuclear microsatellites used in demographic reconstruction in *C. sativa*

**Table S4.** The pairwise differentiation (Fst) among 21 studied populations of *C. sativa* in the South Caucasus computed in FreeNA. The significance was assessed based on 9,999 permutations. All values are in the range of 95% CI

**Table S5.** Regional differences in genetic structure parameters computed for the populations from the South Caucasus

**Table S6.** Posterior probability (PP) obtained for eight tested demographic scenarios of divergence tested in DIYABC-Random Forest. Scenario 1 was indicated as having the highest PP and assumed as the most probable one for populations of *C. sativa* in the Caucasus. Scenario choice was performed in ten replicates ABC-RF analyses based on 140,040 simulated training datasets. The table presents the accuracy metrics of prediction. The number of trees in the constructed random forests was set to 2,000. Standard deviations over the 10 replicate analyses are given in brackets for each metrics.

**Table S7.** The contribution of eight non-correlated bioclimatic variables.

**Fig. S1.** Estimation of the optimal number of genetic clusters of STRUCTURE results (K = 3) based on Evanno’s ΔK method for 22 studied populations of *C. sativa* from the South Caucasus and North Macedonia.

**Fig. S2.** A barplot for K = 3 inferred for 22 studied populations of *C. sativa* from the South Caucasus and North Macedonia based on the Evanno’s ΔK method.

**Fig. S3.** Estimation of the optimal number of genetic clusters of STRUCTURE results (K = 10) based on the mean probability method for 22 studied populations of *C. sativa* from the South Caucasus and North Macedonia.

**Fig. S4.** Estimation of the optimal number of genetic clusters of STRUCTURE results (K = 5) based on the method of Puechmaille (2016) for 22 studied populations of *C. sativa* from the South Caucasus and North Macedonia.

**Fig. S5.** A barplot based on K = 5 inferred for 22 studied populations of *C. sativa* from the South Caucasus and North Macedonia based on an approach of Puechmaille (2016).

**Fig. S6.** Estimation of the optimal number of genetic clusters of STRUCTURE results (K = 10) based on the mean probability method for 21 studied populations of *C. sativa* from the South Caucasus

**Fig. S7.** Estimation of the optimal number of genetic clusters of STRUCTURE results (K = 2) based on Evanno’s ΔK method for 21 studied populations of *C. sativa* from the South Caucasus.

**Fig. S8.** Estimation of the optimal number of genetic clusters for the South Caucasian populations of *C. sativa* (Georgia and Azerbaijan) based on the approach of Puechmaille (2016). The barplot for K = 4 was presented in the main text of this article.

**Fig. S9.** Scenario choice based on the Random Forest approach implemented in DIYABC-Random Forest: (A) Linear discriminant analysis (LDA) projection of the datasets from the training set and observed data on the two first LD axis; (B) prediction power indicating global prior error rate computed using out of bootstrap (OOB error, set as 1000) for random forest versus the number of growing trees in the forest.

plad059_suppl_Supplementary_Material

## Data Availability

All data can be found within the paper and its supporting materials, otherwise available on request from the corresponding author.

## Notes

**Evolution and Diversity. Chief Editor:** Jeremy Beaulieu
