# Supplementary material for: Evolutionary history of Castanea sativa in the Caucasus driven by Middle and Late Pleistocene paleoenvironmental changes
Source: AoB Plants. 2023 Aug 29;15(5):plad059. doi: 10.1093/aobpla/plad059 (PMC10601393; doi:10.1093/aobpla/plad059)
Supplement: plad059_suppl_Supplementary_Material [file plad059_suppl_supplementary_material.docx]

Supplementary information

Evolutionary history of *Castanea sativa* Mill. in the Caucasus driven by Middle and Late Pleistocene paleoenvironmental changes

# **Table S1.** Population identity, location, and genetic diversity parameters for studied populations of sweet chestnut from the South Caucasus and North Macedonia

| Pop | Locality | Latitude | Longitude | Altitude | *N* | *A* | *Pa* | *Ar* | *Ho* | *uHe* | *Fis* | *Null* |
| --- | --- | --- | --- | --- | --- | --- | --- | --- | --- | --- | --- | --- |
| LC1 |  | 41.6843 | 41.8345 | 187 | 30 | 6.67 | 3 | 5.86 | 0.526 | 0.588 | 0.062 |  |
| LC2 | **Lesser**  **Caucasus** | 41.7287 | 42.0781 | 971 | 30 | 6.44 | 2 | 5.51 | 0.548 | 0.594 | 0.029 | 0.051 |
| LC3 |  | 41.9293 | 42.3735 | 465 | 33 | 6.56 | 1 | 5.60 | 0.529 | 0.584 | *0.030** | 0.014 |
| LC4 |  | 41.9581 | 42.7697 | 582 | 31 | 6.67 | 1 | 5.62 | 0.479 | 0.565 | *0.111** | 0.052 |
| **Average** |  |  |  |  |  | **6.59** | **1.75** | **5.65** | **0.521** | **0.583** | **0.058** | **0.045** |
| LR1 | **Likhi**  **Range** | 42.0437 | 43.4988 | 899 | 30 | 5.22 | 1 | 5.60 | 0.474 | 0.546 | *0.086** | 0.050 |
| LR2 |  | 42.1429 | 43.1068 | 365 | 30 | 5.78 | 0 | 4.42 | 0.483 | 0.587 | 0.081 | 0.057 |
| **Average** |  |  |  |  |  | **5.50** | **0.50** | **5.01** | **0.479** | **0.567** | **0.084** | **0.054** |
| WGC1 | **Western**  **Greater**  **Caucasus** | 42.3441 | 43.5264 | 595 | 30 | 5.33 | 2 | 4.62 | 0.506 | 0.530 | 0.025 | 0.040 |
| WGC2 |  | 42.5205 | 43.1896 | 756 | 29 | 5.00 | 0 | 4.58 | 0.492 | 0.539 | 0.018 | 0.055 |
| WGC3 |  | 42.5038 | 43.1462 | 775 | 33 | 5.56 | 0 | 4.80 | 0.529 | 0.546 | 0.033 | 0.017 |
| WGC4 |  | 42.3557 | 42.9991 | 574 | 31 | 4.78 | 0 | 4.20 | 0.503 | 0.554 | 0.029 | 0.077 |
| WGC5 |  | 42.3329 | 42.9421 | 537 | 28 | 6.44 | 5 | 5.64 | 0.583 | 0.562 | 0.021 | 0.018 |
| WGC6 |  | 42.6569 | 42.4341 | 913 | 29 | 5.78 | 0 | 4.92 | 0.437 | 0.533 | *0.131** | 0.077 |
| WGC7 |  | 42.6548 | 42.2216 | 343 | 31 | 6.22 | 0 | 5.39 | 0.482 | 0.518 | *0.073** | 0.024 |
| **Average** |  |  |  |  |  | **5.59** | **1.0** | **4.88** | **0.505** | **0.540** | **0.047** | **0.044** |
| CGC1 | **Central**  **Greater**  **Caucasus** | 42.2226 | 45.3037 | 806 | 18 | 4.67 | 2 | 4.64 | 0.549 | 0.532 | 0.030 | 0.017 |
| CGC2 |  | 41.9472 | 45.9865 | 679 | 30 | 4.44 | 0 | 3.88 | 0.456 | 0.471 | 0.029 | 0.020 |
| CGC3 |  | 41.9326 | 46.0194 | 559 | 27 | 5.00 | 0 | 4.38 | 0.413 | 0.511 | *0.136** | 0.060 |
| CGC4 |  | 41.8854 | 46.2457 | 691 | 22 | 3.56 | 0 | 3.65 | 0.433 | 0.415 | 0.026 | 0.005 |
| **Average** |  |  |  |  |  | **4.42** | **0.50** | **4.14** | **0.463** | **0.482** | **0.055** | **0.026** |
| EGC1 | **Eastern**  **Greater**  **Caucasus** | 41.6796 | 46.6864 | 963 | 33 | 4.56 | 0 | 3.85 | 0.473 | 0.499 | *0.084** | 0.027 |
| EGC2 |  | 41.6194 | 46.6990 | 670 | 31 | 4.11 | 0 | 3.66 | 0.436 | 0.510 | 0.082 | 0.044 |
| EGC3 |  | 41.2976 | 47.1249 | 852 | 29 | 3.56 | 0 | 3.20 | 0.481 | 0.491 | 0.019 | 0.046 |
| EGC4 |  | 40.9883 | 47.9544 | 1058 | 41 | 3.89 | 1 | 3.24 | 0.473 | 0.491 | 0.030 | 0.022 |
| **Average** |  |  |  |  |  | **4.03** | **0.25** | **3.49** | **0.466** | **0.498** | **0.054** | **0.035** |
| MC | **Europe** | 41.7247 | 20.8432 | 953 | 27 | 5.22 | 7 | 4.57 | 0.651 | 0.652 | 0.015 | 0.045 |

Pop – population identity, *N* – number of surveyed individuals, *A* – mean number of alleles, *Pa* – number of private alleles, *Ar* – allelic richness, *Ho* – observed heterozygosity, *uHe* – unbiased estimation of expected heterozygosity, *Fis* – inbreeding coefficient, * – inbreeding is an important factor in the population, *Null* – frequencies of null alleles

# **Table S2.** Priors used for ABC procedure in DIYABC Random Forest for demographic history inferences for *C. sativa*

| **Parameters** | **Distribution** | **Min-Max** | **Mean** | **Shape** | | |
| --- | --- | --- | --- | --- | --- | --- |
| **Genetic parameters** |  |  |  |  | | |
| Mean mutation rate | Log uniform | 1.10^-5^-1.10^-4^ |  |  | | |
| Individual mutation rate | Gamma | 1.10^-7^-1.10^-1^ | Mean mutation rate | | 2 | |
| Mean coefficient P | Uniform | 0.01-1.2 |  |  | | |
| Individual locus coefficient P | Gamma | 0.01-1.5 | Mean coefficient P | | | 2 |
| **Historical parameters** | | | | | | |
| Lineage 1 |  | 10-20000 |  |  | | |
| Lineage 2 |  | 10-1000 |  |  | | |
| Lineage 3 |  | 10-10000 |  |  | | |
| Lineage 4 |  | 10-7000 |  |  | | |
| Lineage 5 |  | 10-20000 |  |  | | |
| *ta* |  | 10-2000 |  |  | | |
| *ra* |  | 0.001-0.999 |  |  | | |
| *t1* |  | 10-2000 |  |  | | |
| *t2* |  | 10-10000 |  |  | | |
| *t3* |  | 10-10000 |  |  | | |
| *t4* |  | 10-10000 |  |  | | |
| Conditions | t1>ta, t2>ta, t2>t1, t3>t2, t4>t3 | | | | | |

# **Table S3.** Characterization of nuclear microsatellites used in demographic reconstruction in *C. sativa*

| **Loci** | **Number of alleles** | ***Ho*** | ***uHe*** | **Fis** | **Null** | **Observed range** | **Reference** |
| --- | --- | --- | --- | --- | --- | --- | --- |
| EMCs15 | 6 | 0.475 | 0.482 | -0.002 | 0.022 | 74-95 | Buck et al. 2003 |
| EMCs2 | 5 | 0.030 | 0.030 | -0.011 | 0.015 | 151-166 | Buck et al. 2003 |
| CsCAT6 | 14 | 0.708 | 0.721 | 0.001 | 0.020 | 144-176 | Marinoni et al. 2003 |
| EMCs13 | 7 | 0.508 | 0.452 | -0.143 | 0.010 | 139-163 | Buck et al. 2003 |
| CsCAT15 | 10 | 0.555 | 0.560 | -0.009 | 0.017 | 121-143 | Marinoni et al. 2003 |
| CsCAT1 | 19 | 0.310 | 0.404 | 0.218 | 0.089 | 175-229 | Marinoni et al. 2003 |
| CsCAT14 | 11 | 0.614 | 0.616 | -0.013 | 0.020 | 133-169 | Marinoni et al. 2003 |
| EMCs22 | 16 | 0.685 | 0.745 | 0.064 | 0.046 | 124-156 | Buck et al. 2003 |
| CsCAT41 | 25 | 0.609 | 0.823 | 0.247 | 0.120 | 206-290 | Marinoni et al. 2003 |
| **Average** | **12.56** | **0.50** | **0.54** | **0.04** | **0.04** | ­— | — |

Marinoni D., Akkak A., Bounous G., Edwards K.J and Botta. R. 2003. Development and characterization of microsatellite markers in *Castanea sativa* (Mill.). Molecular Breeding. 11(2): 127-136.

Buck E.J., Hadonou M., James C.J., Blakesley D and Russell. K. 2003. Isolation and characterization of polymorphic microsatellites in European chestnut (*Castanea sativa* Mill.). Molecular Ecology Notes. 3(2): 239-241.

# **Table S4.** The pairwise differentiation (Fst) among 21 studied populations of *C. sativa* in the South Caucasus computed in FreeNA. The significance was assessed based on 9,999 permutations. All values are in the range of 95% CI

| **Fst** | LC1 | LC2 | LC3 | LC4 | LR2 | LR1 | WGC1 | WGC2 | WGC3 | WGC4 | WGC5 | WGC6 | WGC7 | CGC1 | CGC2 | CGC3 | CGC4 | EGC1 | EGC2 | EGC3 |  |
| --- | --- | --- | --- | --- | --- | --- | --- | --- | --- | --- | --- | --- | --- | --- | --- | --- | --- | --- | --- | --- | --- |
| LC1 |  |  |  |  |  |  |  |  |  |  |  |  |  |  |  |  |  |  |  |  |  |
| LC2 | 0.011 |  |  |  |  |  |  |  |  |  |  |  |  |  |  |  |  |  |  |  |  |
| LC3 | 0.027 | 0.024 |  |  |  |  |  |  |  |  |  |  |  |  |  |  |  |  |  |  |  |
| LC4 | 0.024 | 0.036 | 0.004 |  |  |  |  |  |  |  |  |  |  |  |  |  |  |  |  |  |  |
| LR1 | 0.010 | 0.033 | 0.038 | 0.025 |  |  |  |  |  |  |  |  |  |  |  |  |  |  |  |  |  |
| LR2 | 0.037 | 0.040 | 0.042 | 0.043 | 0.027 |  |  |  |  |  |  |  |  |  |  |  |  |  |  |  |  |
| WGC1 | 0.023 | 0.059 | 0.065 | 0.038 | 0.014 | 0.055 |  |  |  |  |  |  |  |  |  |  |  |  |  |  |  |
| WGC2 | 0.072 | 0.107 | 0.106 | 0.084 | 0.053 | 0.113 | 0.057 |  |  |  |  |  |  |  |  |  |  |  |  |  |  |
| WGC3 | 0.036 | 0.074 | 0.063 | 0.040 | 0.024 | 0.052 | 0.038 | 0.076 |  |  |  |  |  |  |  |  |  |  |  |  |  |
| WGC4 | 0.021 | 0.048 | 0.061 | 0.050 | 0.005 | 0.027 | 0.024 | 0.064 | 0.030 |  |  |  |  |  |  |  |  |  |  |  |  |
| WGC5 | 0.011 | 0.049 | 0.043 | 0.028 | 0.009 | 0.038 | 0.025 | 0.067 | 0.004 | 0.023 |  |  |  |  |  |  |  |  |  |  |  |
| WGC6 | 0.011 | 0.038 | 0.048 | 0.043 | 0.029 | 0.060 | 0.044 | 0.092 | 0.039 | 0.050 | 0.012 |  |  |  |  |  |  |  |  |  |  |
| WGC7 | 0.017 | 0.056 | 0.054 | 0.043 | 0.023 | 0.063 | 0.036 | 0.074 | 0.035 | 0.045 | 0.018 | 0.022 |  |  |  |  |  |  |  |  |  |
| CGC1 | 0.076 | 0.122 | 0.141 | 0.133 | 0.075 | 0.133 | 0.093 | 0.124 | 0.075 | 0.068 | 0.066 | 0.093 | 0.114 |  |  |  |  |  |  |  |  |
| CGC2 | 0.063 | 0.081 | 0.072 | 0.049 | 0.066 | 0.089 | 0.080 | 0.152 | 0.068 | 0.093 | 0.072 | 0.077 | 0.072 | 0.172 |  |  |  |  |  |  | |
| CGC3 | 0.059 | 0.074 | 0.060 | 0.046 | 0.045 | 0.072 | 0.064 | 0.126 | 0.062 | 0.057 | 0.062 | 0.069 | 0.075 | 0.124 | 0.030 |  |  |  |  |  |  |
| CGC4 | 0.073 | 0.115 | 0.121 | 0.115 | 0.102 | 0.151 | 0.116 | 0.146 | 0.116 | 0.125 | 0.088 | 0.064 | 0.104 | 0.145 | 0.107 | 0.125 |  |  |  |  |  |
| EGC1 | 0.070 | 0.107 | 0.098 | 0.080 | 0.073 | 0.123 | 0.070 | 0.133 | 0.070 | 0.104 | 0.072 | 0.073 | 0.071 | 0.119 | 0.042 | 0.050 | 0.100 |  |  |  |  |
| EGC2 | 0.054 | 0.081 | 0.080 | 0.062 | 0.050 | 0.097 | 0.050 | 0.129 | 0.051 | 0.071 | 0.056 | 0.056 | 0.067 | 0.087 | 0.030 | 0.015 | 0.105 | 0.011 |  |  |  |
| EGC3 | 0.051 | 0.082 | 0.093 | 0.080 | 0.066 | 0.118 | 0.073 | 0.110 | 0.079 | 0.079 | 0.067 | 0.064 | 0.063 | 0.114 | 0.069 | 0.034 | 0.104 | 0.051 | 0.043 |  |  |
| EGC4 | 0.151 | 0.164 | 0.150 | 0.135 | 0.116 | 0.148 | 0.158 | 0.174 | 0.135 | 0.140 | 0.142 | 0.168 | 0.163 | 0.196 | 0.136 | 0.106 | 0.266 | 0.148 | 0.135 | 0.152 |  |

# **Table** **S5.** Regional differences in genetic structure parameters computed for the popualtions from the South Caucasus

| Group of populations | *Mean He* | *Mean Ar* | *Mean Fis* | *Mean Fst* |
| --- | --- | --- | --- | --- |
| LC/LR/WGC/CGC/EGC | **0.610/0.599/ 0.561/0.512/0.505** | **5.65/5.01/4.88/4.14/3.49** | 0.041/0.089/-0.002/0.016/-0.010 | 0.024/0.023/0.053/0.101/0.083 |
| LC/WGC | 0.610/0.561 | 5.65/4.88 | 0.041/-0.002 | 0.024/0.053 |
| (LC, WGC)/CGC/(EGC | **0.579/0.512/0.505** | **5.16/4.14/3.49** | 0.014/0.016/-0.010 | 0.050/ 0.101/0.083 |
| (LC, LR, WGC)/(CGC, EGC) | **0.582/0.508** | **5.14/3.81** | 0.026/0.001 | 0.046/0.095 |

Bolded are significant differences at p<0.001; LC = Lesser Caucasus, WGC – West Greater Caucasus, CGC – Central Greater Caucasus, ECG – East Greater Caucasus

# **Table S6.** Posterior probability (PP) obtained for eight tested demographic scenarios of divergence tested in DIYABC-Random Forest. Scenario 1 was indicated as having the highest PP and so was assumed as the most probable one for populations of *C. sativa* in the Caucasus. Scenario choice was performed in ten replicates ABC-RF analyses based on 140,040 simulated training datasets. The table presents the accuracy metrics of prediction. The number of trees in the constructed random forests was set to 2,000. Standard deviations over the 10 replicate analyses are given in brackets for each metrics.

| **ABC-FR run** | **Scenario 1** | **Scenario 2** | **Scenario 3** | **Scenario 4** | **Scenario 5** | **Scenario 6** | **Scenario 7** | **Scenario 8** | **Scenario choice** | **Global error** | **Local error** | **PP** |
| --- | --- | --- | --- | --- | --- | --- | --- | --- | --- | --- | --- | --- |
| 1 | 561 | 278 | 193 | 201 | 168 | 191 | 196 | 212 | 1 | 0.239 | 0.310 | 0.690 |
| 2 | 617 | 286 | 168 | 189 | 142 | 167 | 178 | 253 | 1 | 0.238 | 0.256 | 0.744 |
| 3 | 553 | 251 | 161 | 156 | 172 | 183 | 197 | 327 | 1 | 0.238 | 0.256 | 0.744 |
| 4 | 605 | 244 | 163 | 148 | 178 | 200 | 181 | 281 | 1 | 0.237 | 0.242 | 0.758 |
| 5 | 559 | 263 | 133 | 186 | 174 | 189 | 169 | 327 | 1 | 0.237 | 0.331 | 0.669 |
| 6 | 615 | 285 | 169 | 128 | 126 | 186 | 195 | 296 | 1 | 0.238 | 0.321 | 0.679 |
| 7 | 580 | 290 | 188 | 150 | 157 | 209 | 173 | 253 | 1 | 0.238 | 0.317 | 0.683 |
| 8 | 540 | 255 | 195 | 175 | 178 | 164 | 209 | 284 | 1 | 0.238 | 0.300 | 0.700 |
| 9 | 526 | 246 | 177 | 164 | 208 | 205 | 188 | 286 | 1 | 0.238 | 0.262 | 0.738 |
| 10 | 585 | 264 | 168 | 171 | 154 | 198 | 175 | 285 | 1 | 0.238 | 0.329 | 0.671 |
| **Average** | **574** | **266** | **172** | **167** | **166** | **189** | **186** | **280** | **1** | **0.238** | **0.292** | **0.708** |
| *St. Dev.* | *31.522* | *17.396* | *18.295* | *22.065* | *22.500* | *14.965* | *12.922* | *34.693* |  | *0.0006* | *0.0345* | *0.035* |

# **Table S7.** The contribution of eight non-correlated bioclimatic variables

| Code |  | Bioclimatic variable | LIG | LGM | EH | MH | LH | Current |
| --- | --- | --- | --- | --- | --- | --- | --- | --- |
| Bio01 |  | Annual Mean Temperature | **13.2** | **12.5** | **12.5** | **12.4** | **11.6** | **11.6** |
| Bio03 |  | Isothermality | 0.2 | 0.4 | 0.6 | 0.4 | 0.4 | 0.3 |
| Bio08 |  | Mean Temperature of Wettest Quarter | 1.9 | 0.8 | 1.5 | 1.9 | 0.7 | 1.7 |
| Bio09 |  | Mean Temperature of Driest Quarter | 0.4 | 0.5 | 0.5 | 0.4 | 0.6 | 0.4 |
| Bio15 |  | Precipitation Seasonality | 7.1 | 7.3 | 7.7 | 6.7 | 8.0 | 7.2 |
| Bio18 |  | Precipitation of Warmest Quarter | **55.1** | **55.1** | **55.6** | **54.3** | **55.4** | **56.3** |
| Bio19 |  | Precipitation of Coldest Quarter | **22.2** | **23.3** | **21.5** | **23.8** | **23.4** | **22.5** |
| *AUC* |  |  | *0.975* | *0.973* | *0.974* | *0.975* | *0.973* | *0.973* |


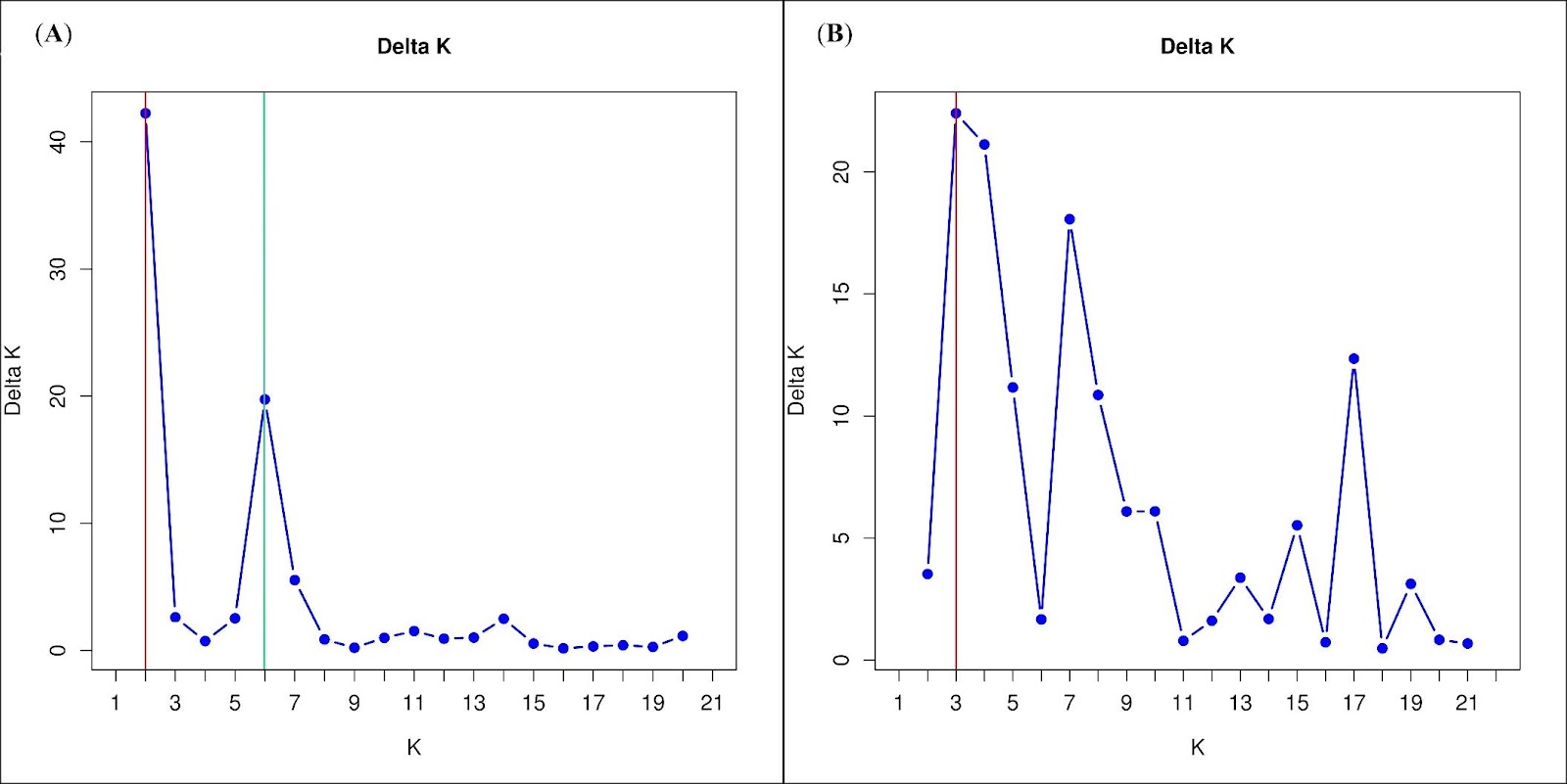


# **Fig. S1.** Estimation of the optimal number of genetic clusters of STRUCTURE results (K=3) based on Evanno’s ΔK method for 22 studied populations of C. sativa from the South Caucasus and North Macedonia


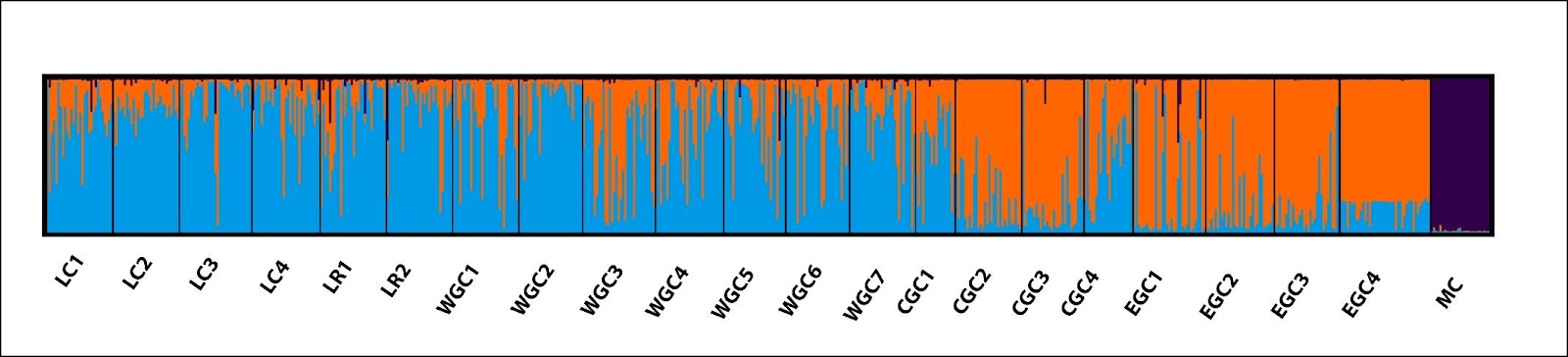


# **Fig. S2.** A barplot for K =3 inferred for 22 studied populations of *C. sativa* from the South Caucasus and North Macedonia based on the Evanno’s ΔK method

#
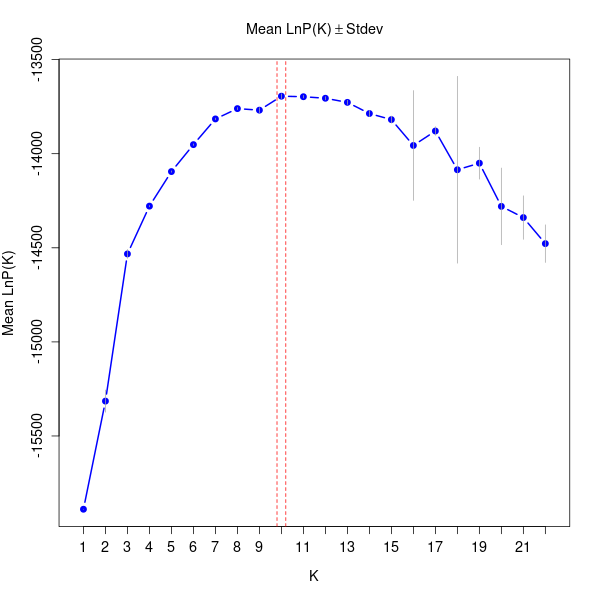


# **Fig. S3.** Estimation of the optimal number of genetic clusters of STRUCTURE results (K=10) based mean probability method for 22 studied populations of *C. sativa* from the South Caucasus and North Macedonia

# **Fig. S4.** Estimation of the optimal number of genetic clusters of STRUCTURE results (K=5) based on method of Puechmaille (2016) for 22 studied populations from the South Caucasus and North Macedonia


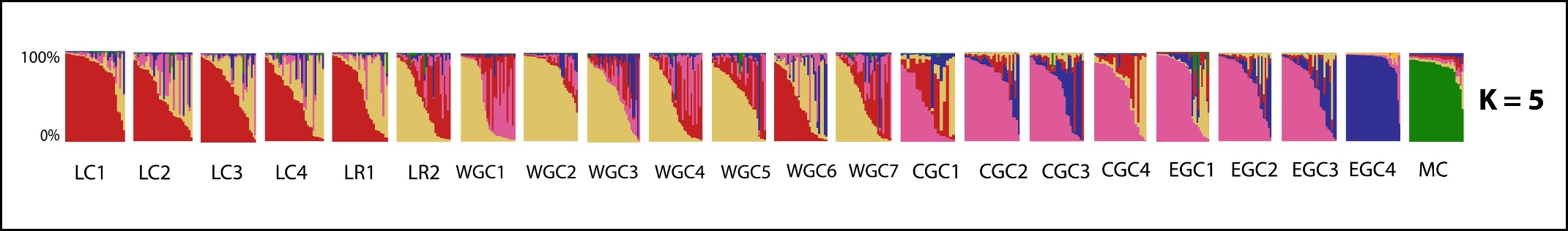


# **Fig. S5.** A barplot based on K=5 inferred for 22 studied populations of *C. sativa* from the South Caucasus and North Macedonia based on an approach of Puechmaille (2016)


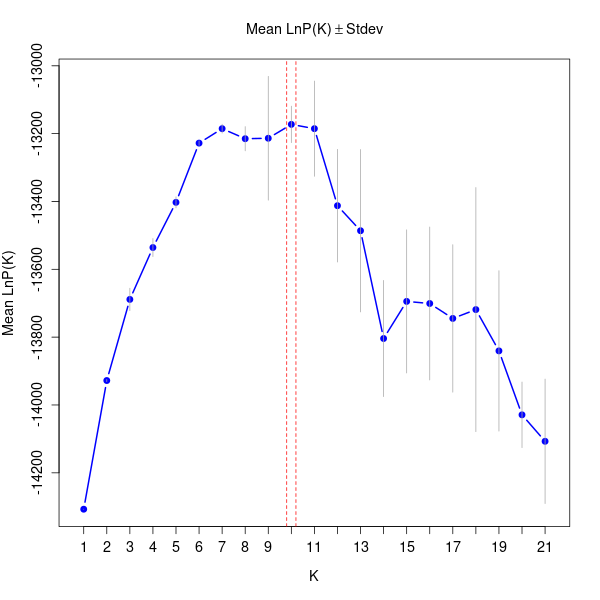


# **Fig. S6.** Estimation of the optimal number of genetic clusters of STRUCTURE results (K=10) based mean probability method for 21 studied populations of *C. sativa* from the South Caucasus

# **
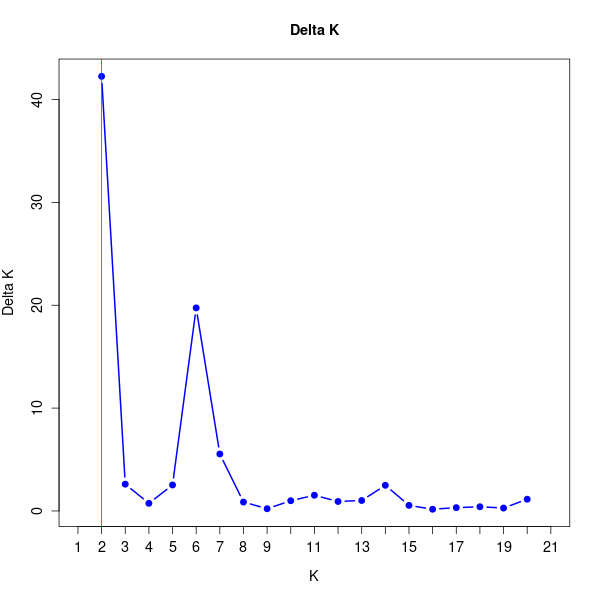
****Fig. S7.** Estimation of the optimal number of genetic clusters of STRUCTURE results (K=2) based on Evanno’s ΔK method for 21 studied populations of C. sativa from the South Caucasus


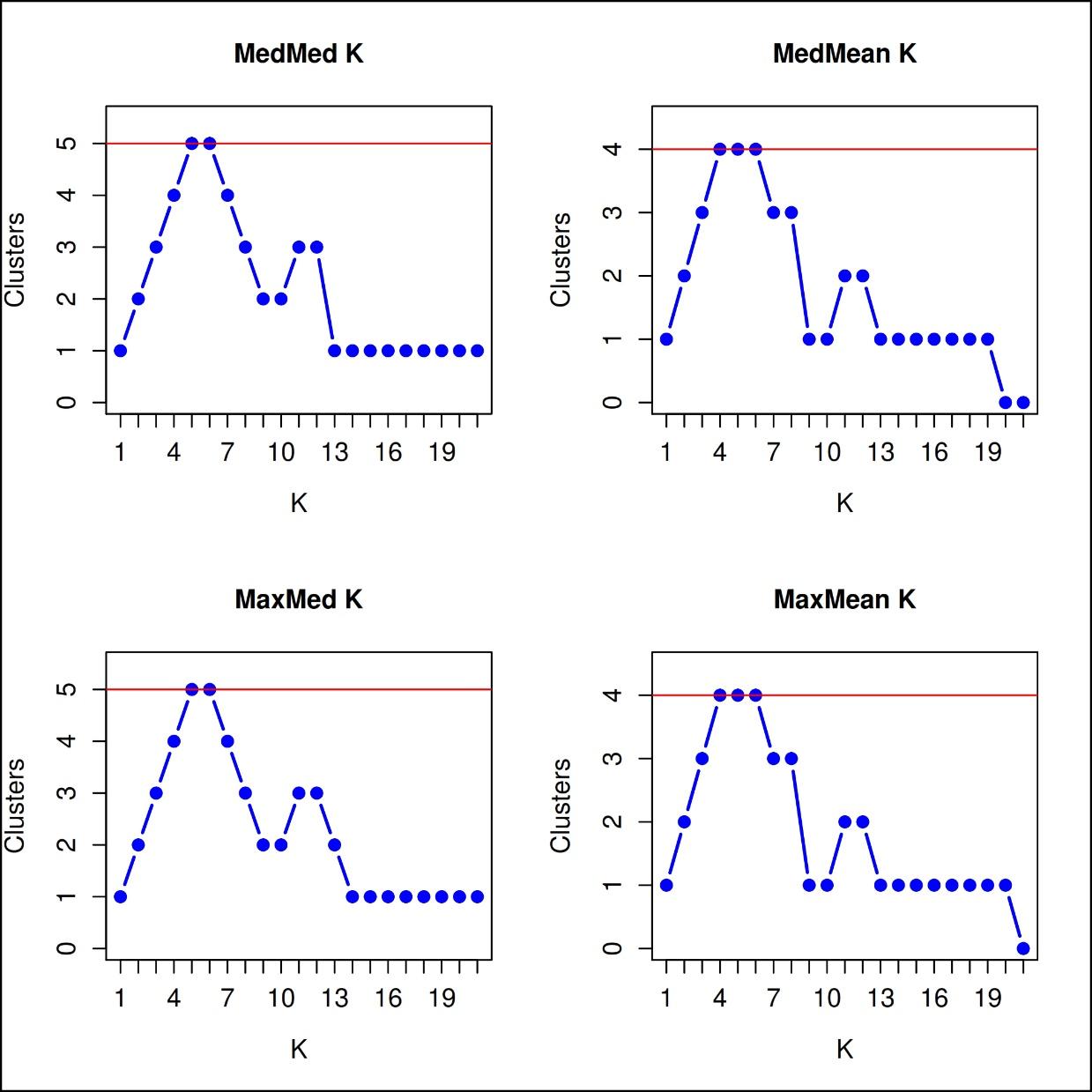


# **Fig. S8.** Estimation of the optimal number of genetic clusters for the South Caucasian populations of *C. sativa* (Georgia and Azerbaijan) based on approach of Puechmaille (2016). The barplot for K=4 was presented in the main text of this article


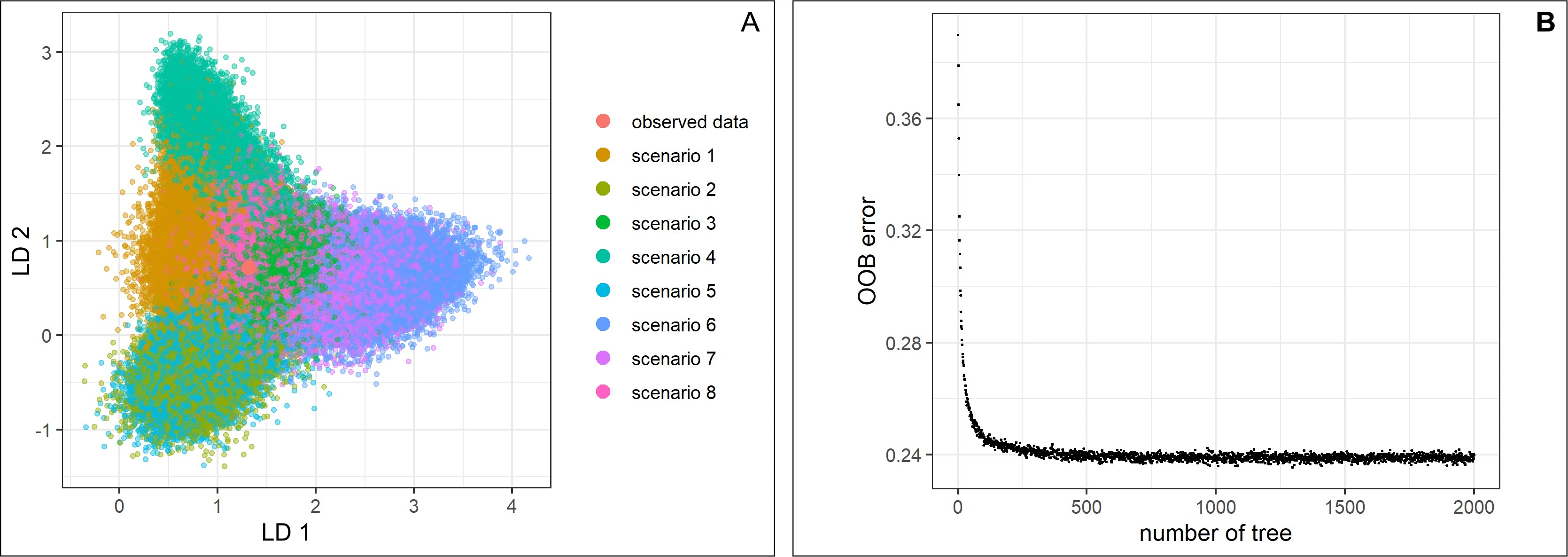


# **Fig. S9.** Scenario choice based on the Random Forest approach implemented in DIYABC Random Forest: (A) Linear discriminant analysis (LDA) projection of the datasets from the training set and observed data on the two first LD axis; (B) prediction power indicating global prior error rate computed using out of bootstrap (OOB error, set as 1000) for random forest versus the number of growing trees in the forest
